# Identification and functional prediction of long noncoding RNAs related to intramuscular fat content in Laiwu pigs

**DOI:** 10.5713/ab.21.0092

**Published:** 2021-06-24

**Authors:** Lixue Wang, Yuhuai Xie, Wei Chen, Yu Zhang, Yongqing Zeng

**Affiliations:** 1 Shandong Provincial Key Laboratory of Animal Biotechnology and Disease Control and Prevention, College of Animal Science and Technology, Shandong Agricultural University, Tai’an, Shandong 271018, China; 2 Department of Immunology, School of Basic Medical Sciences, Fudan University, Shanghai, 200032, China

**Keywords:** Intramuscular Fat, Laiwu Pigs, lncRNAs, mRNAs

## Abstract

**Objective:**

Intramuscular fat (IMF) is a critical economic indicator of pork quality. Studies on IMF among different pig breeds have been performed via high-throughput sequencing, but comparisons within the same pig breed remain unreported.

**Methods:**

This study was performed to explore the gene profile and identify candidate long noncoding RNA (lncRNAs) and mRNAs associated with IMF deposition among Laiwu pigs with different IMF contents. Based on the *longissimus dorsi* muscle IMF content, eight pigs from the same breed and management were selected and divided into two groups: a high IMF (>12%, H) and low IMF group (<5%, L). Whole-transcriptome sequencing was performed to explore the differentially expressed (DE) genes between these two groups.

**Results:**

The IMF content varied greatly among Laiwu pig individuals (2.17% to 13.93%). Seventeen DE lncRNAs (11 upregulated and 6 downregulated) and 180 mRNAs (112 upregulated and 68 downregulated) were found. Gene Ontology analysis indicated that the following biological processes played an important role in IMF deposition: fatty acid and lipid biosynthetic processes; the extracellular signal-regulated kinase cascade; and white fat cell differentiation. In addition, the peroxisome proliferator-activated receptor, phosphatidylinositol-3-kinase-protein kinase B, and mammalian target of rapamycin pathways were enriched in the pathway analysis. Intersection analysis of the target genes of DE lncRNAs and mRNAs revealed seven candidate genes associated with IMF accumulation. Five DE lncRNAs and 20 DE mRNAs based on the pig quantitative trait locus database were identified and shown to be related to fat deposition. The expression of five DE lncRNAs and mRNAs was verified by quantitative real time polymerase chain reaction (qRT-PCR). The results of qRT-PCR and RNA-sequencing were consistent.

**Conclusion:**

These results demonstrated that the different IMF contents among pig individuals may be due to the DE lncRNAs and mRNAs associated with lipid droplets and fat deposition.

## INTRODUCTION

Intramuscular fat (IMF), or marbling, which consists of white spots or stripes of adipose tissue in muscle fiber tracts, is a crucial index affecting meat flavor and tenderness. The IMF is also regarded as a pivotal characteristic to evaluate the quality of pork [1,2]. High IMF is an indicator of symbol of high-grade pork. Thus, to improve the IMF of pork, it is necessary to understand the molecular and cellular mechanisms of fat deposition in muscle. With the development of high-throughput sequencing, genome-wide association studies (GWAS) and RNA sequencing (RNA-seq) have been extensively used in recent years to identify genomic regions and candidate genes associated with a variety of pork traits [3–5]. Furthermore, the appropriate analysis and reliable interpretation of the large datasets obtained by GWAS and RNA-seq is critical for gene expression studies.

Long noncoding RNA (lncRNAs) are vital noncoding RNAs (ncRNAs) that regulate fat accumulation, including the processes of proliferation, differentiation, and hypertrophy of adipocytes [6,7]. Evidence has shown that lncIMF4 regulates porcine intramuscular preadipocytes by attenuating autophagy to inhibit lipolysis [8]. LncRNA IMFlnc1 increases caveolin-1 by sponging miR-199a-5p to promote porcine intramuscular adipocyte adipogenesis [9]. However, fat deposition disorder may also lead to obesity, diabetes, and atherosclerosis [10–12] in humans. RNA-seq performed in humans has determined the molecular regulation mechanisms of fat accumulation between different groups of samples with different genetic backgrounds. For farm animals, RNA-seq conducted using samples obtained from full or half-sibs to control kinship obtains more rigorous data, which will help to elucidate the relatively accurate mechanism of adipogenesis and metabolism, thereby potentially identifying as well as find the novel methods to control fat deposition in meat.

**Figure 1:**
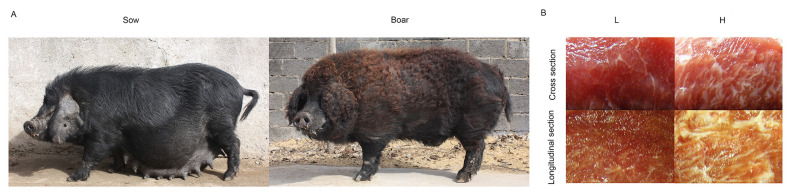
Laiwu pigs [40] and cross and longitudinal sections of pork with different intramuscular fat contents.

Laiwu pigs, an invaluable Chinese indigenous pig breed with high-quality pork, were selected in the present study to investigate the mechanisms of IMF differential expression among individuals [13] (Figure 1A). A previous study comparing Laiwu pigs and Yorkshire pigs has determined the IMF content of Laiwu pigs to be 13.83% [14]. We also found a wide range of variation (2.4% to 17.8%) in the IMF content in a study containing 333 Laiwu pigs [15]. This indicated that the content of IMF varies greatly among Laiwu pig individuals. In addition, few studies were reported on the comparison of gene expression and regulation from same breed, especially for Laiwu pigs. Therefore, based on these previous studies concerning the relationship between gene regulation and fat metabolism, the objective of the present study was to explore and identify valuable candidate lncRNAs and mRNAs associated with IMF accumulation in Laiwu pigs, which may be helpful for breeding pigs to produce high-quality pork. Here, individual Laiwu pigs with high or low IMF (Figure 1B) were identified and selected for analysis in this study. Transcriptomes of *longissimus dorsi* (LD) muscle samples from different Laiwu pig individuals were analyzed using RNA-seq to determine the differentially expressed (DE) lncRNAs and mRNAs from the different phenotypes of the same breed. This research may help to identify genes associated with IMF deposition and their functions in farm animals.

## MATERIALS AND METHODS

### Animals and sample collection

Twenty-nine male 300-d-old Laiwu pigs (approximately 96± 4 kg) reared at the Laiwu pig breeding farm in Jinan, Shandong Province, China, were selected for this study. All pigs were fed the same commercial pig diet and water *ad libitum*. After exsanguination, approximately 100 g of LD muscle from the last rib of each pig was obtained immediately for IMF content determination, RNA preparation, and oil red O staining analysis. For RNA isolation and protein analysis, samples were immediately placed into liquid nitrogen and stored at −80°C until analysis. Samples (1 cm×1 cm×0.5 cm) taken from the cross-section of the LD muscle and the liver were fixed in 4% paraformaldehyde (Solarbio, Beijing, China) for fatty analysis by oil red O staining. The remaining samples were stored at −20°C for the determination of fat content.

### Intramuscular fat content evaluation

The IMF content evaluation was conducted 24 h after slaughter. Based on the “Technical regulation for determination of pork quality (NY/T 821-2004, China)”, IMF content was determined using the Soxhlet petroleum-ether extraction method and expressed as the percentage of fat relative to wet muscle tissue. Specifically, the sample was mashed and dried to a constant weight at 105°C. Following drying, the sample was continuously extracted in petroleum (Caratton, Tianjin, China) for 8 h using a Soxhlet flask. Then, all samples were removed and dried to a constant weight at 80°C. According to the IMF content determined in LD muscle, eight half-sibs of Laiwu pigs were selected and divided into the following two groups: high IMF group and low IMF group, respectively.

### Frozen sections and oil red O staining

Lipid accumulation was visualized by staining cells or tissue sections with the lipid-specific dye, oil red O (Solarbio, China). Tissue was first immersed in 30% isopropanol (Solarbio, China) until it sank to the bottom of the solution. After continuous 14 μm tissue sectioning, sections were fixed in 4% paraformaldehyde solution, washed with tap water for 30 min, and then washed with 60% isopropanol (Hushi, Shanghai, China). Staining was performed by immersing slides in oil red O solution for 30 min. Then, all slides were immersed again in 60% isopropanol, and nuclei were stained in alum hematoxylin (Solarbio, China) for 1 min, washed in distilled water and mounted in aqueous solution.

### Total RNA extraction and library preparation for lncRNA sequencing

Total RNA was extracted with TRIzol Reagent (Invitrogen, Carlsbad, CA, USA) from Laiwu pig muscle samples (stored at −80°C). One gram of total RNA was further treated with RNase-free DNase I (Accurate Biotechnology, Hunan, China) to eliminate genomic DNA contamination according to the manufacturer’s instruction. The integrity and quality of total RNA were evaluated using Agilent 2100 bioanalyzer (Santa Clara, CA, USA) and expressed using the RIN value. The results showed that all samples had RIN values greater than 8.2 (Supplementary Table S1), indicating that all RNA samples met the quality requirement of RNA-seq. All RNA samples were stored at −80°C before further processing.

For RNA-seq, 14 μg of RNA per sample was used as input material for the RNA sample preparations. The RNA was fragmented into 250 to 300 bp fragments after removing the ribosomal RNA using the rRNA Removal Kit (Epicentre, Madison, WI, USA) according to the manufacturer’s instruction. First-strand of cDNA was synthesized using fragmented RNA as a template and random oligonucleotides as primers. After purification, double-stranded cDNA was repaired at the end followed by the addition of A tail, and the DNA was connected with sequencing adapters. AMPure XP Beads were used to screen approximately 350 to 400 bp of cDNA. The USER enzyme (NEB, Ipswich, MA, USA) was used to degrade the second strand of cDNA containing U, and polymerase chain reaction (PCR) amplification was then performed to obtain the library. After library construction, the concentration of the library was measured by a Qubit fluorometer and adjusted to 1 ng/μL. An Agilent 2100 Bioanalyzer was utilized to examine 250 to 300 bp of the acquired library. Finally, the concentration of the cDNA library was verified using quantitative PCR (qPCR). Once the insert size and concentration of the library were greater than 2 nM, the samples were then subjected to sequencing.

### Quality control for raw data and mapping

Raw data (raw reads) in FASTQ format were first processed through in-house perl scripts. In this step, clean data (clean reads) were obtained by removing the following reads: i) reads with 5′ adapter; ii) reads without 3′ adapter or insert sequence; iii) reads with more than 10% N; iv) reads with more than 50% nucleotides with Qphred≤20; and v) reads with poly A/T/G/C. Adapter trimming for the removal of adapter sequences from the 3′ ends of reads was also performed. At the same time, the Q20, Q30, and GC contents of the clean data were calculated. Approximately 93.76% to 94.34% of clean reads were mapped to the reference genome of pigs and 80% of clean reads were uniquely mapped (Supplementary Table S1). All downstream analyses were based on clean data with high quality. Clean reads for each sample were first mapped to a reference genome with HISAT2 software [16]. Reads alignment results were transferred to the program StringTie for transcript assembly.

### Quantification and differential expression analysis

Quantification of the transcripts and genes was performed using StringTie software, and fragments per kilobase of transcript per million mapped reads was obtained. Cuffdiff or edgeR was used for differential expression analysis. The resulting p-values were adjusted using Benjamini and Hochberg’s approach for controlling the false discovery rate. All the transcripts were merged using Cuffmerge software, and then the selected lncRNAs were annotated using Cuffcompare. The reference genome version used for sequence alignment was GCF_000003025.6_Sscrofa11.1. Genes with |log_2_ (fold change)| >0 and padj<0.05 were assigned as DE. There were 17 DE lncRNAs, which included 11 upregulated and 6 downregulated lncRNAs. In addition, there were 180 DE mRNAs, which included 112 upregulated and 68 downregulated mRNAs (Supplementary Figure S1) (p<0.05).

### Gene ontology and Kyoto encyclopedia of genes and genomes enrichment analyses

Gene ontology (GO) analysis describes the functions of genes, and it is divided into the following three parts: molecular functions, biological processes and cellular components. Kyoto encyclopedia of genes and genomes (KEGG) is a comprehensive database integrating genome, chemical, and system functional information [17]. GO seq software was used for GO enrichment analysis. Pathway enrichment analysis was performed using KOBAS (2.0) [18,19]. GO and KEGG enrichment analyses of target genes of DE lncRNAs were performed using the cluster Profiler R package, in which gene length bias was corrected. The enrichment was considered significant when the corrected p-value was less than 0.05.

### Bioinformatics analysis

The high- and low-IMF library preparation and sequencing were performed by Novogene Bioinformatics Technology Corporation. After library preparation, the samples were subjected to Illumina sequencing. A total of 12 Gb of raw data (Supplementary Table S1) for lncRNA-seq analysis were obtained using PE150 (paired-end 150 nt) sequencing.

### Quantitative real time polymerase chain reaction and data analysis

The total RNA was reverse transcribed into cDNA according to the manufacturer’s instructions of the PrimeScript RT reagent Kit with gDNA Eraser (TaKaRa, Dalian, China). All quantitative real time-PCR (qRT-PCR) assays were performed using the Mx3000p Real-Time System (Stratagene, La Jolla, CA, USA). Primers were obtained from Sheng Gong (Shanghai, China), and the sequences are shown in Table 1. The qRT-PCR was performed in triplicate with SYBR Premix Ex Taq (Takara, China) using the following program: 95°C for 30 s; 40 cycles of 95°C for 5 s, 58°C for 30 s, and 72°C for 30 s. To exclude between-run variations, all samples were amplified in triplicates and the means were used for further analysis. Data analyses were performed using the 2^−ΔΔCT^ method.

### Statistical analyses

The statistical significance of differences between the two groups was compared with the unpaired student t-test. The differences were considered statistically significant as: * p< 0.05; ** p<0.01; *** p<0.001.

## RESULTS

### Intramuscular fat content and oil red O staining of samples

**Figure 2:**
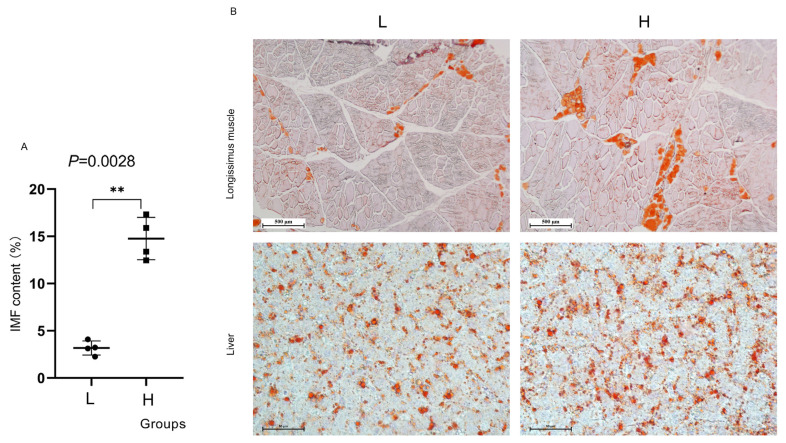
Intramuscular fat (IMF) content and deposition of samples. oil red O staining was performed to detect lipid accumulation in *longissimus dorsi* (LD) muscle and liver. ** p<0.01.

The IMF contents of LD muscle samples from the 29 pigs were determined (Supplementary Table S2), and samples from eight pigs were then selected and divided into two groups for further analysis. The two groups included the high IMF content group (>12%, in terms of H) and the low IMF content group (<5%, in terms of L). Figure 2A shows that the H group had significantly higher IMF content than the L group (p<0.05). To better visualize the differences in muscle and liver transection fat distribution, frozen sections were prepared, and oil red O Staining was performed to elucidate the differences in IMF content in more detail (Figure 2B). The results indicated that compared to the H group, the total area of fat stained by oil red O was larger than that of the L group.

### Gene ontology annotation of differentially expressed genes

**Figure 3:**
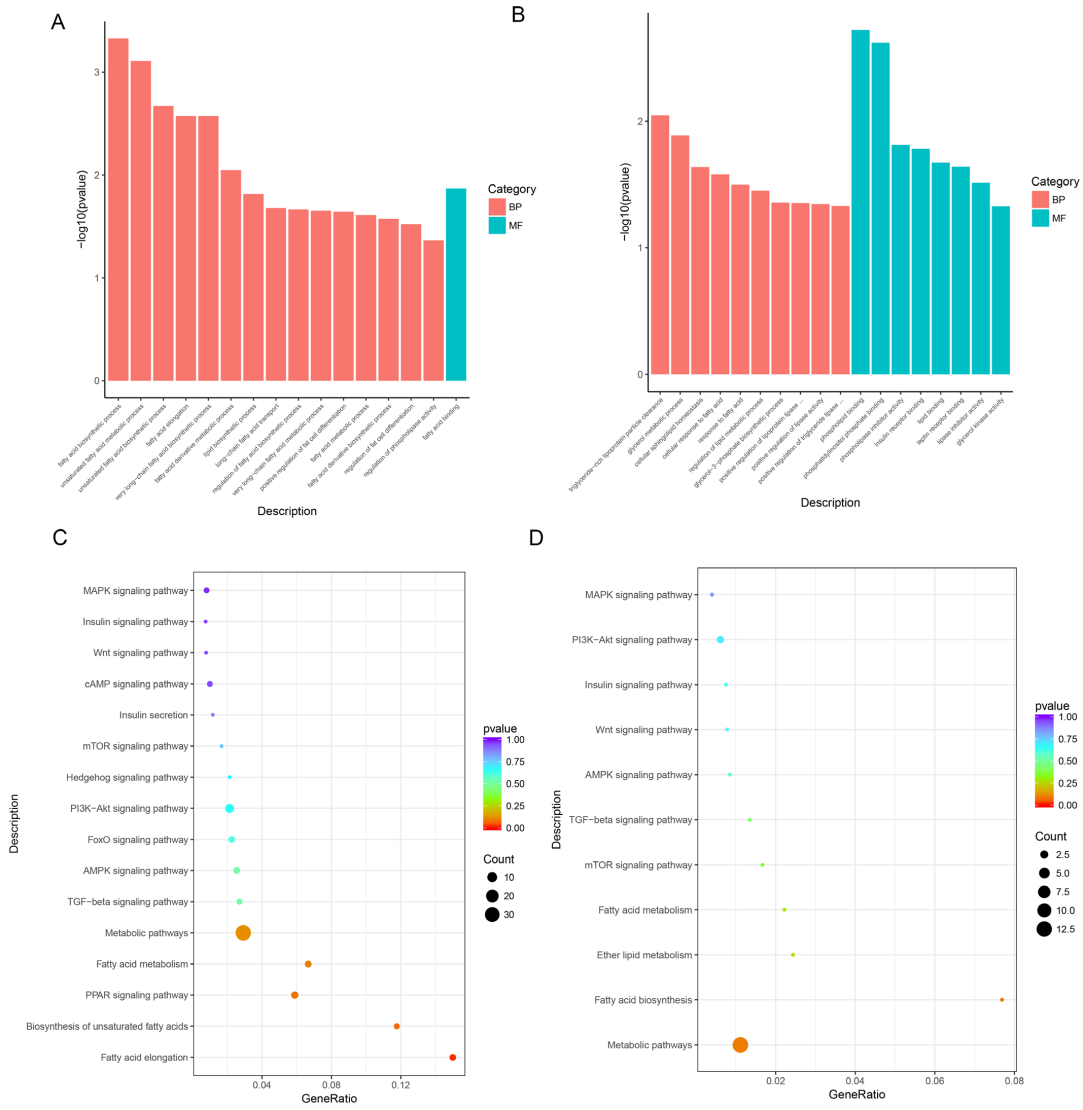
GO terms and KEGG pathways. (A), (C), GO terms and pathways of lncRNAs; (B), (D), GO terms and pathways of mRNAs. GO, gene ontology; KEGG, Kyoto encyclopedia of genes and genomes.

The DE lncRNAs were annotated by GO analysis. As shown in Figure 3, DE lncRNAs were mainly enriched in fatty acid biosynthetic process, unsaturated fatty acid metabolic process, fatty acid metabolic process, and regulation of fat cell differentiation (p<0.05). These genes were also enriched in fatty acid binding molecular functions (Supplementary Table S3). At the same time, the main GO terms of mRNAs related to IMF were significantly enriched in biological processes and molecular functions (p<0.05), including cellular response to fatty acids, cellular sphingolipid homeostasis, insulin receptor binding, and glycerol kinase activity (Supplementary Table S4).

### Kyoto encyclopedia of genes and genomes enrichment analysis of differentially expressed genes

The lncRNA KEGG enrichment analysis demonstrated that 17 DE lncRNAs were enriched in 206 signaling pathways. Among the signaling pathways, the first 15 were listed sorted by p value in Supplementary Table S5. Fatty acid elongation (p = 0.015) and arachidonic acid metabolism (p = 0.019) were the two significantly enriched signaling pathways. In addition, differentially expressed genes (DEGs) were also enriched in signaling pathways associated with fat deposition and lipid metabolism, such as biosynthesis of unsaturated fatty acids, peroxisome proliferator-activated receptor (PPAR) signaling pathway, and steroid biosynthesis (Figure 3C). The mRNA KEGG enrichment analysis demonstrated that 180 DE mRNAs were enriched in 114 signaling pathways (the first 15 were listed sorted by p value in Supplementary Table S6). Similar to lncRNAs, the different mRNAs were also enriched in signaling pathways associated with fat deposition and lipid metabolism (Figure 3D).

### Correlation analysis of lncRNAs and mRNAs

LncRNAs regulate the expression of target genes (mRNAs) through colocation (in *cis*) or coexpression (in *trans*). Intersection analysis of the target genes of DE lncRNAs and the DE mRNAs was performed to elucidate the correlation of lncRNAs and mRNAs. Target gene unions and DE mRNAs of colocation and coexpression were selected for analysis, including two downregulated genes (*NEK10* and *ZFR*) and five up-regulated genes (*ERLEC1*, *RBM47*, *MYOF*, *VWA8*, and *C1orf194*). The intersections (full name) of the target genes of DE lncRNAs and mRNAs are shown in Table 2.

### Expression analysis of IMF-relevant QTLs in Laiwu pigs with low and high IMF contents

**Figure 4:**
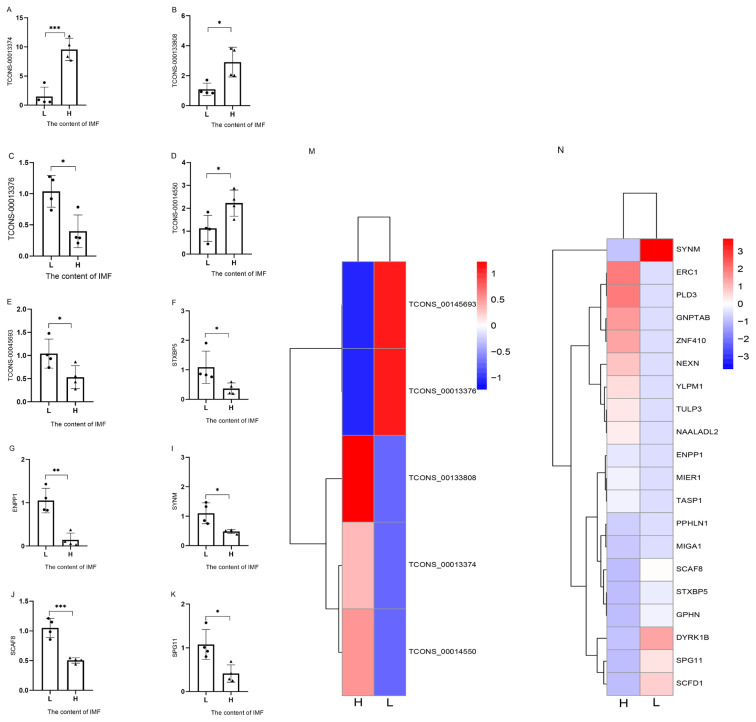
Validation and heatmaps of DE lncRNAs (A–E) and mRNAs (F–K). The DEGs in LD tissues between Laiwu pigs with high and low IMF contents was verified by qRT-PCR. DE, differentially expressed; DEGs, differentially expressed genes; LD, *longissimus dorsi*; IMF, intramuscular fat; qRT-PCR, quantitative real time polymerase chain reaction; *ERC1*, ELKS/RAB6-interacting/CAST family member 1; *PLD3*, phospholipase D family member 3; *GNPTAB*, N-Acetylglucosamine-1-phosphate transferase subunits α and β; *ZNF410*, zinc finger protein 410; *NEXN*, Nexilin F-actin binding protein; *YLPM1*, YLP motif containing 1; *TULP3*, TUB like protein 3; *NAALADL2*, N-Acetylated α-linked acidic dipeptidase like 2; *MIER1*, MIER1 transcriptional regulator; *TASP1*, taspase 1; *PPHLN1*, periphilin 1; *MIGA1*, mitoguardin 1; *GPHN*, gephyrin; *DYRK1B*, dual specificity tyrosine phosphorylation regulated kinase 1B; *SCFD1*, sec1 family domain containing 1. * p<0.05; ** p<0.01, *** p<0.001.

To confirm the differences in gene transcription levels between the lowest marbling grade and highest marbling grade samples, we examined the expression of IMF-associated lncRNAs and mRNAs based on the pig quantitative trait locus (QTL) database (https://www.animalgenome.org/cgi-bin/QTLdb/SS/index). In total, 25 DEGs in LD muscle were detected, including five lncRNAs (TCONS_00145693, TCONS_00013374, TCONS_00133808, TCONS_00013376, and TCONS_00014550) and 20 mRNAs (listed in the heatmap in Figure 4M, 4N). The expression of five lncRNAs and mRNAs was confirmed by qRT-PCR. The qRT-PCR analysis showed the expression of lncRNAs (Figure 4A–4E, TCONS_00013374, TCONS_00133808, TCONS_00014550, TCONS_00013376, and TCONS_00145693) and mRNAs (Figure 4F–4J, syntaxin binding protein 5 [*STXBP5*], ectonucleotide pyrophosphatase phosphodiesterase 1 [*ENPP1*], synemin [*SYNM*], SR-related CTD associated factor 8 [*SCAF8*], and spastic paraplegia gene 11 [*SPG11*]), which were screened based on pig QTL databases (Figure 4). The qRT-PCR results for these genes were consistent with the RNA-seq results.

## DISCUSSION

The Laiwu pig is an excellent breed raised in China with a high IMF content. Several previous studies concerning IMF content have been reported; however, most of these studies were performed to compare the difference between two breeds of pigs [14,20,21] to investigate DEGs associated with IMF deposition. However, in animal production, we found that IMF content varies vastly among individual Laiwu pigs. Among the 29 LD samples tested, the high IMF content could be achieved 13.93%, while the low content was only 2.17%, which was confirmed by pork marbling (Figure 1) and oil red O staining (Figure 2). Our results were similar to the results that a wide range of variation (2.4% to 17.8%) in the IMF trait of Laiwu pig individuals reported in a previous study [12]. Although all types of cells contain the same genome, they have different structures and behaviors due to the cell and tissue specificity of gene expression. All cells in multicellular organisms are derived from a single cell, which differentiates in response to external or internal cell signals and gradually establishes different gene expression patterns to exhibit different functions. Identical twins are not identical, and similarly, clonal microbial cells or Laiwu pigs (our samples in this article) differ in many aspects even when grown simultaneously in a common environment. This phenomenon may be caused by cell-to-cell heterogeneity in gene expression [22]. Cell-to-cell heterogeneity in lipid droplet production results in differences in lipid storage [23,24], which may be responsible for differences in IMF content among individuals of the same breed, even if pigs are under the unified environment. We hypothesize that the significant difference in IMF content among individuals may be regulated by cell-to-cell heterogeneity in candidate gene expression in fat deposition. In the present study, all pigs were selected from the same breed, same sex, and similar weight to allow candidate genes to be more reliable, thereby excluding unreliable factors identified due to different breeds or different feed conditions. To the best of our knowledge, our study is the first attempt to elucidate this phenomenon, which would help to provide new perspectives into the mechanism of IMF accumulation in pigs.

With the development of high-throughput sequencing, an increasing number of studies have indicated that lncRNAs regulate fat deposition processes by affecting epigenetic regulation, transcription, posttranscriptional levels and protein translation processes [7,25,26]. Fat deposition in animals is primarily determined by the proliferation and hypertrophy of adipocytes. Evidence has shown that lncRNA Gm15290 promotes PPARγ-induced fat accumulation and contributes to body weight gain in mice by sponging miR-27b [27]. LncRNA IMFNCR promotes intramuscular adipocyte differentiation in poultry by acting as a ceRNA to be combined with miR-128-3p and miR-27b-3p [6]. Therefore, it is of great significance to explore DE lncRNAs and the mechanisms of IMF deposition.

In GO and KEGG analyses, the DEGs were enriched in the processes of fatty acid elongation and biosynthesis, of which the PPAR, phosphatidylinositol-3-kinase-protein kinase B (PI3K-Akt), and mammalian target of rapamycin signaling pathways were significantly highly enriched. These pathways have been demonstrated to be associated with the processes of adipogenesis, preadipocyte differentiation, and transformation of brown and white fat cells [28–31]. Although the functions of these DE lncRNAs and mRNAs are still unclear, these lncRNAs and mRNAs might regulate fat deposition by these signaling pathways.

To explore the candidate genes related to IMF deposition, intersection analysis of the target genes of DE lncRNAs and mRNAs was performed. Seven genes were obtained, which were both lncRNA target genes and mRNAs (*ERLEC1*, *RBM47*, *MYOF*, *VWA8*, *C1orf194*, *NEK10*, and *ZFR*). One of the genes is zinc finger RNA binding protein (*ZFR*), which is an ancient protein in eukaryotic genomes. Snail family transcriptional repressor 2 (*SNAI2*, also known as *SLUG*), a C2H2-type zinc finger transcription factor, is expressed in white adipose tissue in humans and regulates adipocyte differentiation by affecting the expression of PPARγ [32]. *GATA2* and *GATA3* belong to the C2C2-type zinc finger protein subfamily and are negative regulators of the preadipocyte-to-adipocyte transition. Previous studies have confirmed that *GATA2* and *GATA3* downregulate the expression of adipocyte differentiation markers such as PPARγ and CCAAT enhancer binding protein α (C/EBPα) [33,34]. Although several studies have suggested that the other six genes (*ERLEC1*, *RBM47*, *MYOF*, *VWA8*, *C1orf194*, and *NEK10*) are associated with fat content, there are many studies on the regulation of *ZFR* in fat deposition. Our sequencing results also provided a new direction to explore the functions of these genes in regulating fat accumulation as each of them may act as the next *ZFR* in the future.

In addition, 20 protein-coding genes were obtained from the results of DEGs based on the pig QTL database as follows: *SCAF8*, *STXBP5*, *ENPP1*, *SPG11*, *SYNM*, *TULP3*, *ERC1*, *PPHLN1*, *GNPTAB*, *DYRK1B*, *PLD3*, *MIGA1*, *NEXN*, *MIER1*, *SCFD1*, *GPHN*, *ZNF410*, *YLPM1*, *NAALADL2*, and *TASP1* (the full name was shown in Figure 4). Many of these genes have been shown to be associated with fat deposition. The *ENPP1* has an important role in adipogenesis processes. The *ENPP1* expression in adipose tissue is increased in obese patients with insulin resistance [35], and *ENPP1* is also considered both a biomarker and a novel potential therapeutic target to improve adipose tissue function and systemic glucose metabolism. *TULP3*, an essential regulator of ciliary GPCR (G protein–coupled receptors) entry, is critical for adipogenesis. It has been shown that the expression levels of *TULP3* correlate with adipogenic potential [36]. In addition, *DYRK1B*, a member of the DYRK family, mediates the transcriptional activation of PPARγ and C/EBPα in adipogenic differentiation [37]. The number of DEGs was decreased by combining sequencing data with the pig QTL database. The function of the screened differential genes by this method in fat deposition may be valuable.

It is relatively hard for lncRNAs to be applied in livestock production. First, the function of lncRNA target genes is the starting point to study the function of lncRNAs. Gene chips should be constructed to select animals expressing or not expressing specific genes. Second, interference or overexpression of candidate lncRNAs or mRNAs can be used to confirm the functions of these genes, such as lncRNA-siRNA, lncRNA-antisense oligodeoxynucleotides, and overexpression vectors [38]. CRISPR/Cas9 has become a powerful method to alter the structure of genes or the genome of many organisms [39], and it may be used to identify whether these genes function due to the specific sequence or the transcription. Because most of these methods are largely used in cells or small animals, such as mice, the protocol and dosage for pigs, which are relatively large animals compared with mice, need to be confirmed. In the future, additives that affect candidate lncRNAs or mRNAs may be identified, which may be help to improve the IMF content of pork. Therefore, finding a reliable candidate lncRNA or mRNA is a basic and crucial step.

## CONCLUSION

In conclusion, this is the first report on lncRNAs in the LD muscle of half-sibling relationship Laiwu pigs with high and low IMF contents analyzed based on the RNA-seq approach. Seventeen DE lncRNAs and 180 mRNAs were identified, and the target genes of lncRNAs and mRNAs were enriched in the fat accumulation process. In addition, two methods (based on the pig QTL database and correlation analysis of the targets of lncRNAs and mRNAs) were performed to screen the DE lncRNAs and mRNAs from the sequencing data, and several genes screened by these two methods have been demonstrated to be related to fat deposition. Seven lncRNA target genes, namely, *ERLEC1*, *RBM47*, *MYOF*, *VWA8*, *C1orf194*, *NEK10*, and *ZFR* were identified and validated as associated with the IMF content. Therefore, we hypothesize that these genes may have important roles in the IMF deposition process. In future research, various technical methods will be performed to identify the functions and mechanisms of these DEGs to select pigs with high IMF content. The present study provides a reference for lncRNAs and mRNAs that can be used for biomedical research related to intramuscular fat accumulation in the future. However, this is only the first step in exploring the mechanisms of fat deposition. Further understanding of the molecular mechanisms controlling adipogenesis is crucial to improving pork quality.

## Figures and Tables

**Table 1:** Primers for real-time polymerase chain reaction analysis

Gene	Sequence (5′-3′)
*TCONS_00145693*	F: CGTGAGCTGTGGTGTAGGTTGC R: CCAGGCTAGGGGTCCAATCGG
*TCONS_00013374*	F: AGAAGGTGGTCCTCTGCTGTGG R: CCTTTTCTCTGGCTCCCTTTTCCC
*TCONS_00133808*	F: ATAGGGTCACTACAGGGTCCACAG R: TCCAGCACCACACTCCAAGGG
*TCONS_00013376*	F: GGTCCTCTGCTGTGGGAGTCTC R: ATTTGGCTTGACAGTGGGTGATGG
*TCONS_00014550*	F: ATCCATCACCAACACCAGCTCAAC R: TCCTGGTCTTCCTTCTCGGCATC
*SCAF8*	F: GCAGTCAGCAGCATTTCCTTGAAC R: TCTCCTCTTCCACTCCATCTTGCC
*STXBP5*	F: CCAGAGTGTGAACAGGCACCAC R: AGGATGAGACTGAGGAGGAATGGG
*ENPP1*	F: GCATAGGTAGTGGGCAGCAAGG R: CAAGCGTGGGAGGCAGTCAAC
*SPG11*	F: CCAGTGCTCAGAGGTGCCAAAC R: AGACATGAGGCCAGGACACTAAGG
*SYNM*	F: GGTAGCAGACAGCAGCAGAACAC R: CCAAGCACCACGGTCAGACAC
*β-actin*	F: AATCCTGCGGCATCCACGAAAC R: CAGCACCGTGTTGGCGTAGAG

**Table 2:** Intersection analysis of the target genes of differentially expressed lncRNAs and mRNAs

LncRNA ID	LncRNA expression	Gene ID	mRNA expression
TCONS_00013376	Down	*NEK10* (NimA-related kinase 10)	Down
TCONS_00013376	Down	*ZFR* (Zinc finger RNA binding protein)	Down
TCONS_00166724	Up	*ERLEC1* (Endoplasmic reticulum lectin 1)	Up
TCONS_00111510	Up	*RBM47* (RNA binding motif protein 47)	Up
TCONS_00166724	Up	*MYOF* (Myoferlin)	Up
TCONS_00133808	Up	*VWA8* (Von willebrand factor A domain containing 8)	Up
TCONS_00170056	Up	*C1orf194* (Chromosome 1 open reading frame 194)	Up

## SUPPLEMENTARY MATERIAL

Characteristics of the lncRNAs (A) and mRNAs (B) expression levels.

**Supplementary Table S1**. Summary of raw reads after quality control and mapping to the reference genome

**Supplementary Table S2**. The information of the 29 Laiwu pigs

**Supplementary Table S3**. The GO terms of DE lncRNA enriched in

**Supplementary Table S4**. The GO terms of DE mRNA enriched in

**Supplementary Table S5**. The DE lncRNAs KEGG enrichment analysis

**Supplementary Table S6**. The DE lncRNAs KEGG enrichment analysis
