# Supplementary material for: Identification and functional prediction of long noncoding RNAs related to intramuscular fat content in Laiwu pigs
Source: Anim Biosci. 2021 Jun 24;35(1):115–25. doi: 10.5713/ab.21.0092 (PMC8738936; doi:10.5713/ab.21.0092)
Supplement: Supplementary file 1 [file ab-21-0092-suppl.pdf]

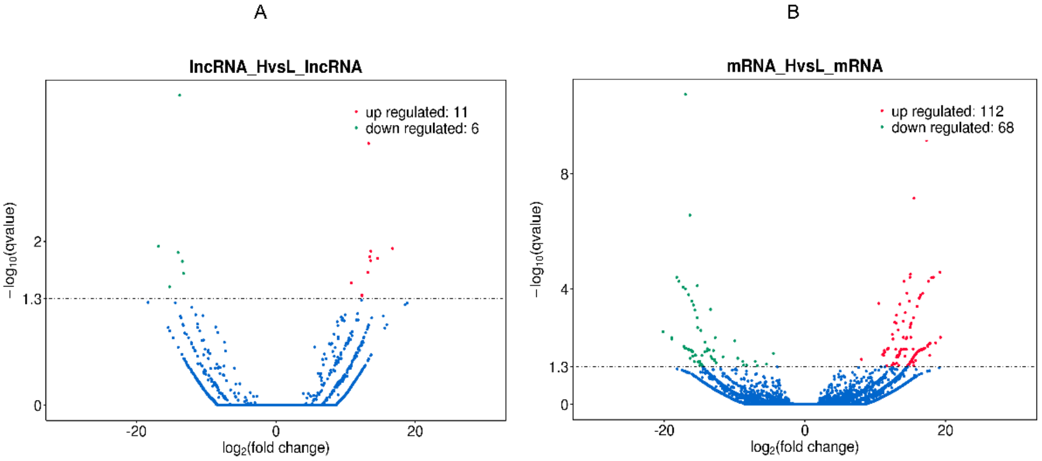

Figure 1 Characteristics of the lncRNAs (A) and mRNAs (B) expression levels.

Table 1

Summary of raw reads after quality control and mapping to the reference genome

Sample	RIN value	Raw reads	Clean reads	Q20 (%)	Q30 (%)	GC content (%)	Total mapped	Uniquely mapped
H8	8.70	83681052	82688222	97.65	93.4	55.21	77617415 (93.87%)	71111569 (86%)
H20	8.20	82438026	81120734	97.78	93.71	55.17	76436184 (94.23%)	68114702 (83.97%)
H21	9.20	87989636	86300212	97.84	93.91	54.92	81416966 (94.34%)	73085143 (84.69%)
H6	8.60	83017054	82107994	97.63	93.32	55.94	77247927 (94.08%)	69941573 (85.18%)
L13	8.40	87912524	86337236	97.48	93.08	55.71	80951280 (93.76%)	72719814 (84.23%)
L22	8.50	83722398	82720782	97.81	93.87	56.69	77658084 (93.88%)	69785246 (84.36%)
L23	8.50	82491086	81265912	97.77	93.72	56.5	76191870 (93.76%)	67562346 (83.14%)
L3	8.30	83590522	81914094	97.88	94.02	56.26	76820759 (93.78%)	68183609 (83.24%)

Table 2

The information of the 29 Laiwu pigs.

Earmarks	Weight (kg)	IMF content (%)	Earmarks of father	Earmarks of mother
42505	***97.5***	***13.93***	***961***	***286***
47507	***99.5***	***13.38***	***1467***	***860***
41505	***96.5***	***13.27***	***11***	***44***
43203	***96.5***	***12.45***	***579***	***436***
44403	99	10.92	1467	1094
46407	98.8	10.74	579	72
48903	98.4	10.41	61	742
46005	98	10.07	11	128
43607	98	10.06	1467	1264
45005	97.7	9.81	1235	130
46001	97.5	9.25	11	128
48905	92	8.86	61	742
46609	95.1	7.6	11	972
43907	95.1	7.59	409	874
43603	94.9	7.44	1467	1264
45609	94.7	7.26	961	764
41903	94.7	7.25	1467	160
46803	94.5	7.08	1467	336
41107	94.4	6.99	1467	348
47405	94.2	6.83	11	226
48303	95.5	6.77	961	1304
47501	93	5.8	1467	860
47403	93.5	5.52	11	226
45503	93.5	4.35	1467	36
46609	***92***	***4.31***	***11***	***972***
45605	***93***	***4.29***	***961***	***764***
44305	***97***	***4.23***	***579***	***606***
43609	***98.5***	***3.23***	***1467***	***1264***
47603	94	2.17	487	1462

Table 3

The GO terms of DE lncRNA enriched in.

GO_accession	Description	Term_type	*P*
GO:0006633	fatty acid biosynthetic process	biological_process	0.00047
GO:0033559	unsaturated fatty acid metabolic process	biological_process	0.00078
GO:0006636	unsaturated fatty acid biosynthetic process	biological_process	0.0021
GO:0030497	fatty acid elongation	biological_process	0.0027
GO:0042761	very long-chain fatty acid biosynthetic process	biological_process	0.0027
GO:1901568	fatty acid derivative metabolic process	biological_process	0.0090
GO:0005504	fatty acid binding	molecular_function	0.014
GO:0008610	lipid biosynthetic process	biological_process	0.015
GO:0015909	long-chain fatty acid transport	biological_process	0.021
GO:0042304	regulation of fatty acid biosynthetic process	biological_process	0.022
GO:0000038	very long-chain fatty acid metabolic process	biological_process	0.022
GO:0045600	positive regulation of fat cell differentiation	biological_process	0.023
GO:0006631	fatty acid metabolic process	biological_process	0.025
GO:1901570	fatty acid derivative biosynthetic process	biological_process	0.027
GO:0045598	regulation of fat cell differentiation	biological_process	0.030
GO:0010517	regulation of phospholipase activity	biological_process	0.043

Table 4

The GO terms of DE mRNA enriched in.

GO_accession	Description	Term_type	*P*
GO:0005543	phospholipid binding	molecular_function	0.0019
GO:1901981	phosphatidylinositol phosphate binding	molecular_function	0.0024
GO:0071830	triglyceride-rich lipoprotein particle clearance	biological_process	0.0090
GO:0006071	glycerol metabolic process	biological_process	0.013
GO:0004859	phospholipase inhibitor activity	molecular_function	0.015
GO:0005158	insulin receptor binding	molecular_function	0.016
GO:0008289	lipid binding	molecular_function	0.021
GO:1990460	leptin receptor binding	molecular_function	0.023
GO:0090156	cellular sphingolipid homeostasis	biological_process	0.023
GO:0071398	cellular response to fatty acid	biological_process	0.026
GO:0055102	lipase inhibitor activity	molecular_function	0.031
GO:0070542	response to fatty acid	biological_process	0.032
GO:0019216	regulation of lipid metabolic process	biological_process	0.035
GO:0046167	glycerol-3-phosphate biosynthetic process	biological_process	0.044
GO:0051006	positive regulation of lipoprotein lipase activity	biological_process	0.044
GO:0060193	positive regulation of lipase activity	biological_process	0.045
GO:0061365	positive regulation of triglyceride lipase activity	biological_process	0.047
GO:0004370	glycerol kinase activity	molecular_function	0.047

Table 5

The DE lncRNAs KEGG enrichment analysis.

Signaling Pathways	*P*
Fatty acid elongation	0.015
Arachidonic acid metabolism	0.019
Biosynthesis of unsaturated fatty acids	0.070
PPAR signaling pathway	0.084
Steroid biosynthesis	0.097
Fatty acid metabolism	0.098
Metabolic pathways	0.12
Linoleic acid metabolism	0.18
Sphingolipid metabolism	0.29
TGF-beta signaling pathway	0.52
AMPK signaling pathway	0.53
FoxO signaling pathway	0.61
PI3K-Akt signaling pathway	0.65
Hedgehog signaling pathway	0.67
mTOR signaling pathway	0.76

Table 6

The DE lncRNAs KEGG enrichment analysis.

Signaling Pathways	*P*
Metabolic pathways	0.096
Fatty acid biosynthesis	0.098
Glycerophospholipid metabolism	0.13
Ether lipid metabolism	0.27
ABC transporters	0.28
Fatty acid metabolism	0.29
Inositol phosphate metabolism	0.35
mTOR signaling pathway	0.36
TGF-beta signaling pathway	0.43
Phosphatidylinositol signaling system	0.45
AMPK signaling pathway	0.59
Wnt signaling pathway	0.62
Insulin signaling pathway	0.63
PI3K-Akt signaling pathway	0.70
MAPK signaling pathway	0.84
